# Using three-dimensional equieffective dose mapping to audit a methodology for calculating permitted doses for head and neck reirradiation^☆^

**DOI:** 10.1016/j.phro.2025.100867

**Published:** 2025-11-17

**Authors:** David Nash, Sarah Muscat, Chelmis M. Thiong’o, Eliana Vasquez Osorio, Antony L. Palmer

**Affiliations:** a Department of Medical Physics, Portsmouth Hospitals University NHS Trust, Portsmouth, UK; b Division of Cancer Sciences, The University of Manchester, Manchester, UK; c Radiotherapy-Related Research, The Christie NHS Foundation Trust, Manchester, UK; d Department of Medical Physics and Biomedical Engineering, University College London, London, UK

**Keywords:** Reirradiation, Spinal cord, Brainstem, Head and neck

## Abstract

**Background and purpose:**

Reirradiation is increasingly common, but accounting for prior dose to the spinal cord and brainstem is challenging. This study compares a simple isodose-based method of previous dose compensation with voxel-wise equivalent dose in 2 Gy fractions (EQD_2/2_) dose mapping to assess accuracy and safety.

**Materials and methods:**

Ten head and neck reirradiation cases were retrospectively reviewed. During planning, original dose distributions were mapped onto the reirradiation computed tomography scan. Isodoses within the cord and brainstem were used to segment dose-level substructures, with cumulative doses kept within defined tolerances. Following 3D EQD_2/2_ mapping, the same cases were audited by recalculating cumulative maximum doses. Cumulative doses from both methods were compared.

**Results:**

Retrospective EQD_2/2_ analysis confirmed all cumulative doses were within tolerance, with up to 9.7 Gy difference between the two methods. In two cases, cord and brainstem doses approached tolerance.

**Conclusions:**

A simple, isodose-based approach to spinal cord and brainstem reirradiation tolerance calculation has been shown to be safe when retrospectively compared with voxel-wise EQD_2/2_ mapping. This method can be implemented in any planning system capable of generating contours from isodose lines, offering a practical alternative where advanced EQD_2/2_ dose accumulation software is unavailable.

## Highlights

• Simple isodose method supports safe head and neck reirradiation.
• Dose-based segmentation used to guide re-treatment planning.
• Voxel-wise equivalent dose conversion confirmed reliability.
• Voxel-wise dose mapping confirmed agreement.
• No neurological side effects observed in audited patient cohort.

## Graphical abstract

**Figure:**
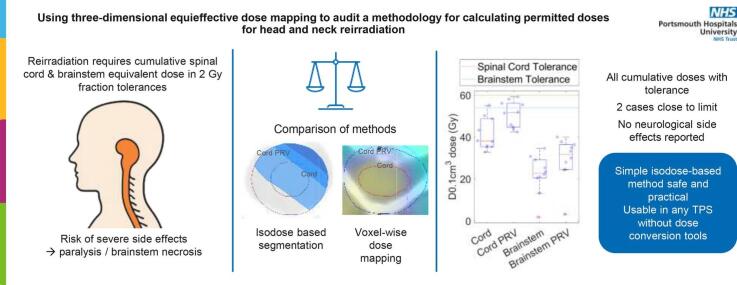

## 1 Introduction

Radiotherapy forms treatment for approximately 80 % of head and neck patients [1]. Highly conformal techniques, such as intensity modulated radiotherapy (IMRT), and volumetric arc therapy (VMAT) are commonly used as a treatment option, with sharp dose gradients shaping the high dose to the target and achieving good organ at risk (OAR) sparing. Even though survival has increased, e.g., 5-year survival by 11–22 % [2], around 40 % of patients experience locoregional recurrence or new disease close to previously irradiated tissues [3]. In this case the treatment options may be surgery, chemotherapy, immunotherapy or potentially repeat radiotherapy, also known as reirradiation. Reirradiation rates are unclear, although ranges from 1 % [4] to ∼10 % [5] have been reported. These cases present a challenge in the clinic as careful consideration of OAR tolerances needs to be undertaken to ensure the patient does not experience debilitating side effects.

Serial OARs, including the spinal cord and brainstem, are of special concern as their damage can cause severe life limiting side effects. For the spinal cord this may include paralysis and myelopathy over a timespan of six months to two years and for the brainstem may include necrosis leading to brain damage. Recovery of normal tissues between treatments is an active area of research and with small numbers of patients, tolerances are often poorly defined.

Spinal cord tolerances to reirradiation is one of the better studied topics. Nieder et al [6,7] reported that no radiation myelopathy was noted for patients where the gap between treatments was greater than 6 months and the equivalent dose in 2 Gy fractions with α/β = 2 Gy (EQD_2/2_) is less than 120 Gy BED_2_ (biologically equivalent dose), so long as the BED_2_ for each course is less than 98 Gy_2_. The risk increased from 0 to 3 % after cumulative BED_2_ of 135.5 Gy_2_. A more recent study by Nieder et al [4] used a revised tolerance of 25 % recovery after 6 months and 50 % recovery after 12 months and no side effects were reported although they did note that there was still the risk of side effects in surviving patients. Another study by Sminia et al. [8] suggested that cumulative doses of 125 % and 172 % of single-course cord tolerance values were safe. More work exists for stereotactic ablative body radiotherapy (SABR) and stereotactic radiosurgery (SRS) [9,10], but this work is of limited applicability to conventional fractionation schedules. A suitable tolerance for the cord would be acumulative EQD_2/2_ tolerance for cord of 60 Gy, exceptionally allowing a cumulative EQD_2/2_ of up to 67.75 Gy. The higher tolerance was used in situations where unacceptable compromise of target coverage would have been required to achieve the lower tolerance value. These values are equivalent to cumulative BED_2_ tolerances of 120–135.5 Gy_2_, derived from Nieder et al [6,7].

Brainstem tolerances are more poorly defined, with work generally limited to brain cancer. One study suggested that reirradiation tolerances values of 73.8 Gy EQD_2/2_ is safe [9], whilst the BRIOche trial assumes a recovery factor of 25 % [11]. Based on the uncertainty, assuming a 0 % recovery factor for brainstem would be appropriate.

This study presents a simple technique for computing re-treatment tolerances for cord and brainstem. It was developed at a time when three-dimensional conversion of dose into EQD_2/2_ or BED equivalent was not available. Basing the re-treatment tolerance values for cord and brainstem only on the maximum dose received by these structures in the first treatment would have been unnecessarily restrictive, as the regions of maximum dose would not necessarily have coincided between treatments. Instead, a technique that considered the spatial distribution of dose in these organs from the initial treatment was developed. This technique requires no particular software capabilities other than the capacity to register two scans, to propagate the dose distribution from one to the other and convert isodoses to contours.

With the advent of treatment planning systems that have the capacity to undertake 3D dose conversion into EQD2, it is now possible to retrospectively audit the combined EQD2 dose distribution of a cohort of patients historically planned using this methodology, to test the validity of this technique. The neurological side effects reported by patients after reirradiation were also investigated for this group of patients.

## 2 Materials and methods

### 2.1 Patient selection and original treatment planning

Ten patients who received reirradiation for head and neck cancer were selected from the archives at Portsmouth Oncology Centre, UK. We selected patients that received a radical dose of radiation in both treatment courses, which in our institution ranges between 55 Gy in 20 fractions to 65.1 Gy in 30 fractions. We selected type 1 reirradiations as per the ESTRO reirradiation classification retreated anytime between 2016 and 2024 [12]. Based on this, ten patients met the criteria, details of which are shown in Table S1. The exact fractionation patterns are reported, along with EQD_2/2_ doses. Elective dose irradiation at a lower dose, usually at 54 Gy, was used in 60 % of patients. A variety of head and neck sites were treated, some bilateral or ipsilateral. Planning computed tomography scan (pCT), contours, dose distribution and plan of the previous treatment course were collected. We planned all the reirradiation cases in Pinnacle (Philips, NL) after contouring in ProSoma (Medcom, France), and they were approved by four different Oncologists.

Ethics approval was not required for this study according to the NRES ethics approval decision tool, as the original work was conducted as part of clinical practice and the audit was classified and registered as a service evaluation as per internal hospital processes.

All ten patients experienced locoregional recurrence of their previously treated cancer, with varying intervals. Table S1 in the supplementary materials gives a summary of the patient characteristics for this study. The gap between treatments varied from 8 months to 63 months.

### 2.2 Reirradiation planning strategy

The reirradiation CT scan (rCT), was non-rigidly fused with the previous treatment scan, pCT, and the previous dose mapped to the rCT, using either ProSoma or Pinnacle. The registration was manually checked for feasibility, and to account for registration uncertainties, a conservative approach with dose constraints was implemented. This included checking for any deformation of bone, consistency between musculature in the neck and agreement of organs at risk. From this propagated dose, isocontours corresponding to previous dose thresholds were created. Using the Boolean operators in the TPS for the organ planning risk volume (PRV), sub-contours were created from the maximum dose decreasing in 5–10 Gy steps down to 10–20 Gy. The maximum dose for each sub-contour was extracted. This was then converted to EQD_2/2_ using the linear quadratic equation:-

(1) $$\mathrm{EQ}D_{2/2}=D+\frac{d+\frac{\alpha }{\beta }}{2+\frac{\alpha }{\beta }}$$

where D is the total dose and d is the fraction dose for each segment and α/β = 2 Gy. The permitted reirradiation EQD_2/2_ for each segment was calculated by subtracting the delivered dose from the maximum permitted cumulative dose of 60 Gy [6] using

(2) $$\mathrm{Segment}\;\mathrm{maximum}\;\mathrm{re}-irradiation\;EQD_{2/2}=60Gy-delivered\;EQD_{2/2}$$

**Fig. 1:**
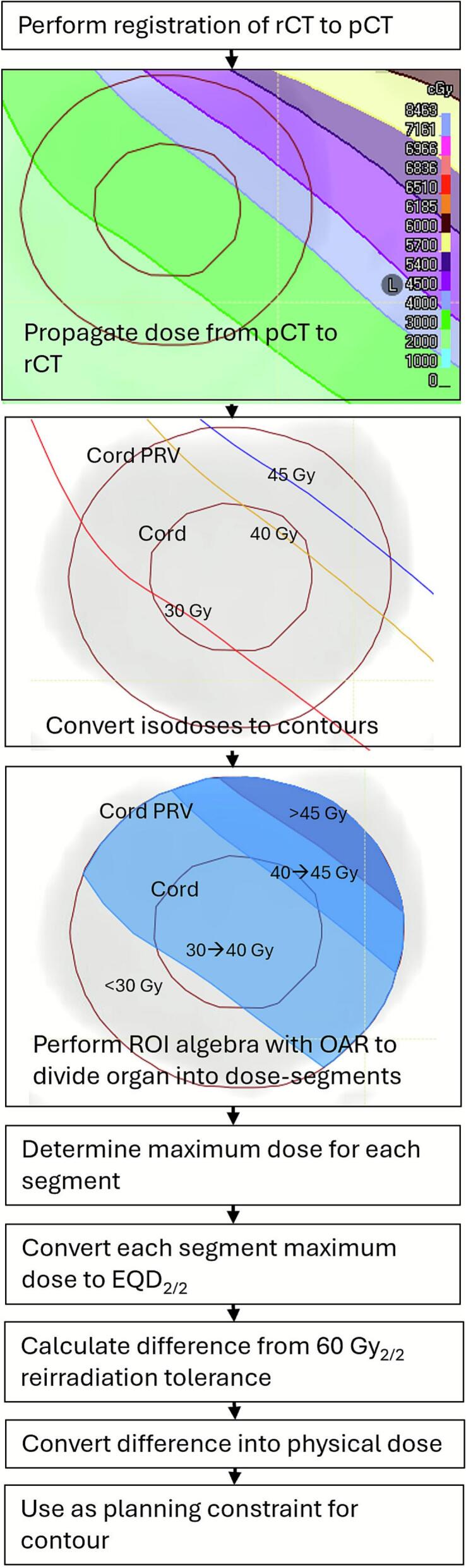
Schematic illustration of the process of creating the contours. This shows the initial deformable registration and the dose propagation. Isodoses were then converted into contours and the intersection of these with the OAR was used to create sub-structures within the OAR. The maximum dose in each organ segment was then used to calculate a physical dose constraint for that segment such that the cumulative EQD_2/2_ within the segment did not exceed the organ tolerance. These constraints were used in the planning of the re-irradiation.

**Fig. 2:**
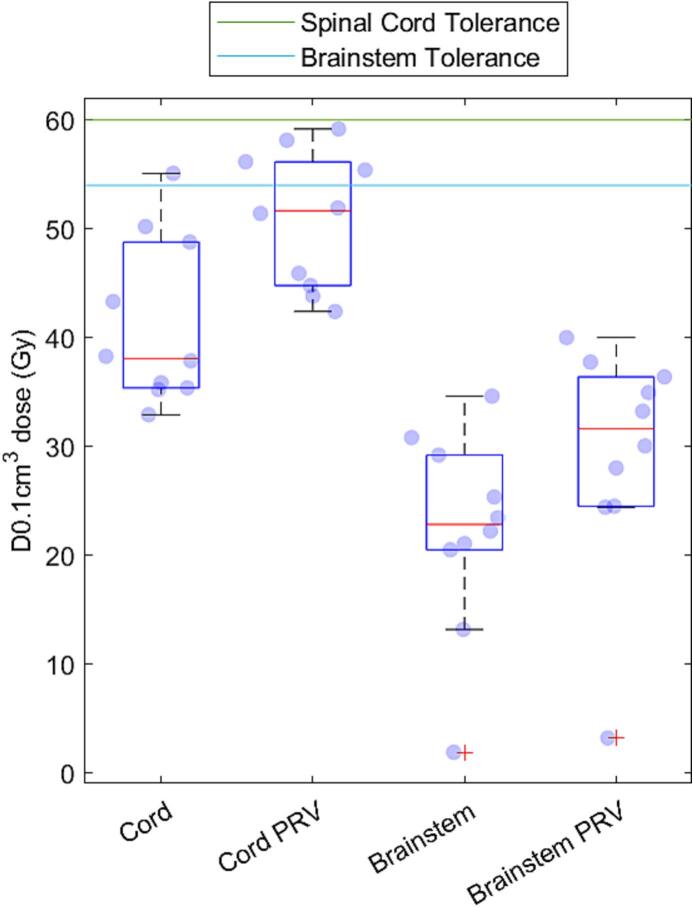
Example of a segmented cord PRV structure (red line) for the spinal cord (blue line), showing three segmented dose levels with different permitted reRT doses, up to 43.5 Gy in orange, 39.1 Gy in cyan, 34.0 Gy in magenta. (For interpretation of the references to colour in this figure legend, the reader is referred to the web version of this article.)

This value was then converted back to physical dose using re-organisation of equation (1) and used for optimisation of the reirradiation plan. The process is illustrated in Fig. 1, with an example in Fig. 2 of a segmented cord PRV.

If brainstem was close to the reirradiation volume, the same process was used for calculating reirradiation doses using a cumulative EQD_2/2_ dose constraint of ≤54 Gy. This was based on standard single-course constraint at Portsmouth Oncology Centre (UK) and assumes no recovery.

If the brainstem was further from the reirradiation site (i.e. a dose considerably less than tolerance and unlikely to be exceeded, recognising it is likely the inferior margin of the OAR that will receive this dose as no nasopharynx cases were in the cohort), then a simpler calculation based only on the organ point dose maximum was used. Three patients did not have any assessment of brainstem dose due to distance from the site.

### 2.3 Reirradiation independent audit

We retrospectively audited the combined distribution for the patients, to determine whether the combined EQD_2/2_ dose received by cord and brainstem was within the cumulative dose constraints. This was performed using the voxel based EQD2 conversion tools in RayStation 2024A (RaySearch, Sweden).

For this audit all treatment data for both courses was imported to RayStation. The pCT and rCT were non-rigidly registered, and the registration verified for integrity. Both dose cubes were then converted to EQD2. The normal tissues were converted using α/β = 3 Gy and the spinal cord and brainstem were converted using α/β = 2 Gy. Note that PRVs were not used for conversion purposes. Following this, the previous EQD2 dose was mapped to the rCT using the deformation map that had been determined and both doses were accumulated voxel-by-voxel. The D0.1 cm^3^ (maximum dose to 0.1 cm^3^) of the brainstem and spinal cord were extracted. Note that in the original method, because of the small size of the dose segments, the maximum dose metric was used for each segment to calculate reirradiation tolerance and estimated cumulative dose. However, for the audit, we have chosen to extract the near-maximum dose (D0.1 cm^3^), as Raystation does not report on an absolute maximum dose to an organ.

At the time of planning these cases, the estimated cumulative maximum dose to the organ PRV was calculated by summing the maximum dose to each segment from the first and second treatment and reporting the dose to the segment with the highest combined dose. This was compared with the D0.1 cm^3^ value to the PRV derived from the RayStation audit.

### 2.4 Audit of reported side effects

A retrospective audit of the clinical notes was also undertaken. The clinical post-treatment letters were reviewed by an experienced Physicist for any report of neurological side effects, such as paralysis, evidence of myelopathy, brain damage or loss of function. All notes were originally written by the Oncologist, although no formal grading criteria was followed. It is not possible from this method to categorise side effects according to any established scoring system, as the data has not been captured. In addition, many patients have died since their reirradiation, so it would not be possible to contact the patients for additional information.

## 3 Results

### 3.1 Reirradiation audit results

Fig. 2 shows the cumulative doses for the spinal cord and brainstem, and their PRVs, derived from the retrospective audit. The cumulative dose to brainstem was below organ tolerance in all cases. The spinal cord PRV came close to tolerance on two patients, with the highest being below 60 Gy. Dose to spinal cord was lower because reirradiation constraints were applied to the PRV rather than to the organ.

Table 1, Table 2 show the comparison of maximum dose to the two organs as calculated by the two methods. Note that only 7 patients for the brainstem were considered. The spinal cord PRV maximum dose was on average 1.2 ± 5.9 Gy less on the RayStation calculation than the value estimated from the original method, whilst the brainstem PRV was 7.3 ± 9.1 Gy less. In one patient the achieved dose to cord was higher than the permitted optimal dose of ≤60 Gy EQD_2/2_, but within the mandatory tolerance of ≤67.75 Gy EQD_2/2_.

**Table 1:** Maximum dose statistics for spinal cord PRV for the 10 patients showing the estimated EQD_2/2_ from the original method, the RayStation EQD_2/2_ and the difference between the two values.

Patient	Original EQD_2/2_ (Gy)	RayStation EQD_2/2_ (Gy)	Difference (Gy)
1	59	59.7	0.7
2	42.2	51.9	9.7
3	67.4	60.4	−7.0
4	57.3	56.4	−0.9
5	50	44.9	−5.0
6	58.8	59.6	0.8
7	47.8	46.4	−1.4
8	58.1	49.4	−8.7
9	47.8	53.5	5.7
10	49	43.2	−5.8

**Table 2:** Maximum dose statistics for brain stem PRV for the 10 patients showing the estimated EQD_2/2_ from the original method, the RayStation EQD_2/2_ and the difference between the two values.

Patient	Original EQD2/2 (Gy)	RayStation EQD2/2 (Gy)	Difference (Gy)
1	47.8	33.7	−14.1
2	49.8	37.2	−12.6
3	25.6	20.3	−5.2
4	42.9	49.6	6.7
5	Brainstem not considered		
6	42.4	41.3	−1.1
7	45.9	28.4	−17.5
8	Brainstem not considered		
9	Brainstem not considered		
10	33.8	26.5	−7.3

### 3.2 Audit of reported side effects

The audit of clinical notes showed minimal reported neurological side effects. Only three patients had recorded neurological side effects in the notes. Patient 4 showed non-specific brain deterioration, believed to be linked to disease progression reported two months post-reirradiation, whilst patient 6 showed memory loss. Patient 10 showed a facial palsy of unknown origin a month after reirradiation. It was not recorded in the notes of any of the patients whether the effects noted were due spinal cord or brainstem injury.

## 4 Discussion

In this work we present our reirradiation planning strategy, a technique that can be adopted in the majority of treatment planning systems to compensate for previous dose received by spinal cord and brainstem. We audited this technique for ten reirradiation patients, using modern registration algorithms to map the previous treatment dose to the retreatment CT, converting both treatment doses to EQD_2/2_ and accumulating the dose on the rCT to determine if our technique was effective in constraining the cumulative dose to cord and brainstem. Our work demonstrated that none of the patients received cumulative doses to these organs that exceeded tolerance, indicating that this technique was effective for achieving this goal. It is acknowledged that for the brainstem the we assumed no repair, which may have been excessively conservative, as it is likely that some repair would have occurred.

In terms of the side effect audit, this is recognised as being very limited in scope, as side effects may not have been consistently reported, and follow up may have been limited. However it was assumed that any major side effects occurring during the follow-up period would have been documented. As reported, patient 6 reported some memory loss. However, in this case, this was only reported after a subsequent third course of orthovoltage and electron radiotherapy to the scalp which occurred approximately two years following the second irradiation. It is unlikely that this reported effect is a result of the head and neck reirradiation.

It is well known that different deformation algorithms [14] and even different settings in a single algorithm [15] may give different results when propagating dose, therefore there may be significant errors in dose propagation and there is no gold standard for dose accumulation available. In this study it is reassuring that the maximum doses calculated by the two methods are similar, despite the fact that two different systems have been used for the registration of CT scans and subsequent dose mapping. This suggests that there is low uncertainty in dose propagation for these organs, presumably because they are unlikely to vary significantly between the primary and reirradiation. However, this may not be the case for organs that are more variable over time. In such cases, it is advised to perform dose accumulation using multiple registrations with different settings to gain an understanding of uncertainties in cumulative dose estimation. Our approach of considering maximum dose to a PRV rather than to the organ itself also mitigates against uncertainties in does propagation and accumulation.

There have been many papers on reirradiation tolerances for spinal cord and brainstem, but few publications offering suggestions on how to achieve these in the clinic. The BRIOche trial recommends a similar approach for gliomas [11]. This is believed to be one of the first studies demonstrating the direct application of a reirradiation dose tolerance in a patient-specific manner, with an audit of its effectiveness including side-effect data. In addition, this technique could be directly applied to any treatment planning system in any clinic. Therefore, by adopting the techniques presented in this paper a centre could adopt a comprehensive approach for spinal cord and brainstem reirradiation which does not compromise care to the patient. If a centre chose to use a different organ tolerance to that presented here, it would be simple to adapt this methodology to the tolerance adopted by the centre. Other body sites where this could be of use is brain, with various structures such as eyes and optic structures being of interest. The audit has shown this technique to be effective, and so it can be potentially used for other serial organs in any part of the body.

A similar piece of work was performed by Hague & Chiu where rigid registration was used to segment the cord, brainstem and other organs in a case report of 3 patients [13]. They assumed 50 % recovery, but this still ended up with the threshold of 120 Gy_2_ BED for the cord but allowed up to 127 Gy_2_ for the brainstem. The 50 % recovery was determined through clinical experience of the centre’s oncologists. However, they did not validate their approach using EQD_2/2_ summation and just report a similar method to what we present here. They did show that of the three patients in the case report that 2/3 patients survived for more than 5 years post treatment with no toxicities greater than grade 2. They did consider more organs, and in the PTV itself, however this work is a case report of 3 patients compared to our 10 and no auditing of the doses after treatment was conducted.

A further limitation of this work is the small number of patients. Although head and neck reirradiation is it is the fourth leading tumour site for reirradiation [16,17], over the course of several years, the numbers in our institution have not been very high. Nevertheless, as our audit has shown that our original methodology led to cumulative doses for all patients which are generally considered to be acceptable, it is reasonable to assume that the same conclusions would have been reached with a larger study population. However, it should be noted that the audit of side effects is very limited due to the small study size and further larger studies are required to correlate toxicities with cumulative dose in the reirradiation setting.

It should also be noted that the reirradiation planning strategy proposed in this study is designed for serial-type organs where maximum, or near-maximum, dose constraints are generally considered. It cannot easily be applied to parallel organs, where volumetric dose constraints, such as mean organ dose, are generally considered.

This paper describes a method for calculating reirradiation tolerances that considers the spatial distribution of dose within an organ in the primary irradiation, rather than simply considering the maximum dose previously received by the organ. The retrospective audit of this method using tools now available to us in Raystation has shown that this method is robust. An additional audit of recorded side effects in this cohort of patients after reirradiation was also undertaken and none of the patients audited appear to have had radiation-induced late effects. The technique itself is simple to implement in the clinical setting and does not require advanced treatment planning features such as 3D EQD_2/2_ dose conversion and mapping, which might not be available in all radiotherapy centres.

## Declaration of generative AI and AI-assisted technologies in the manuscript preparation process

During the preparation of this work the author(s) used ChatGPT in order to shorten the abstract. After using this tool/service, the author(s) reviewed and edited the content as needed and take(s) full responsibility for the content of the published article.

## Data statement

All data supporting this study are provided in the manuscript and supplementary materials accompanying this paper. No funding or grants were awarded for this work.

## CRediT authorship contribution statement

**David Nash:** Conceptualization, Methodology, Visualization, Investigation, Formal analysis, Writing – original draft. **Sarah Muscat:** . **Chelmis M. Thiong’o:** Methodology, Conceptualization, Visualization, Supervision, Writing – original draft. **Eliana Vasquez Osorio:** Conceptualization, Methodology, Writing – review & editing. **Antony L. Palmer:** Writing – review & editing, Supervision.

## Declaration of competing interest

The authors declare that they have no known competing financial interests or personal relationships that could have appeared to influence the work reported in this paper. Eliana Vasquez Osorio is a Guest Editor for this journal and was not involved in the editorial review or the decision to publish this article.
