# Supplementary material for: Using three-dimensional equieffective dose mapping to audit a methodology for calculating permitted doses for head and neck reirradiation
Source: Phys Imaging Radiat Oncol. 2025 Nov 17;36:100867. doi: 10.1016/j.phro.2025.100867 (PMC12702338; doi:10.1016/j.phro.2025.100867)
Supplement: Supplementary Data 1 [file mmc1.pptx]

## Slide 1
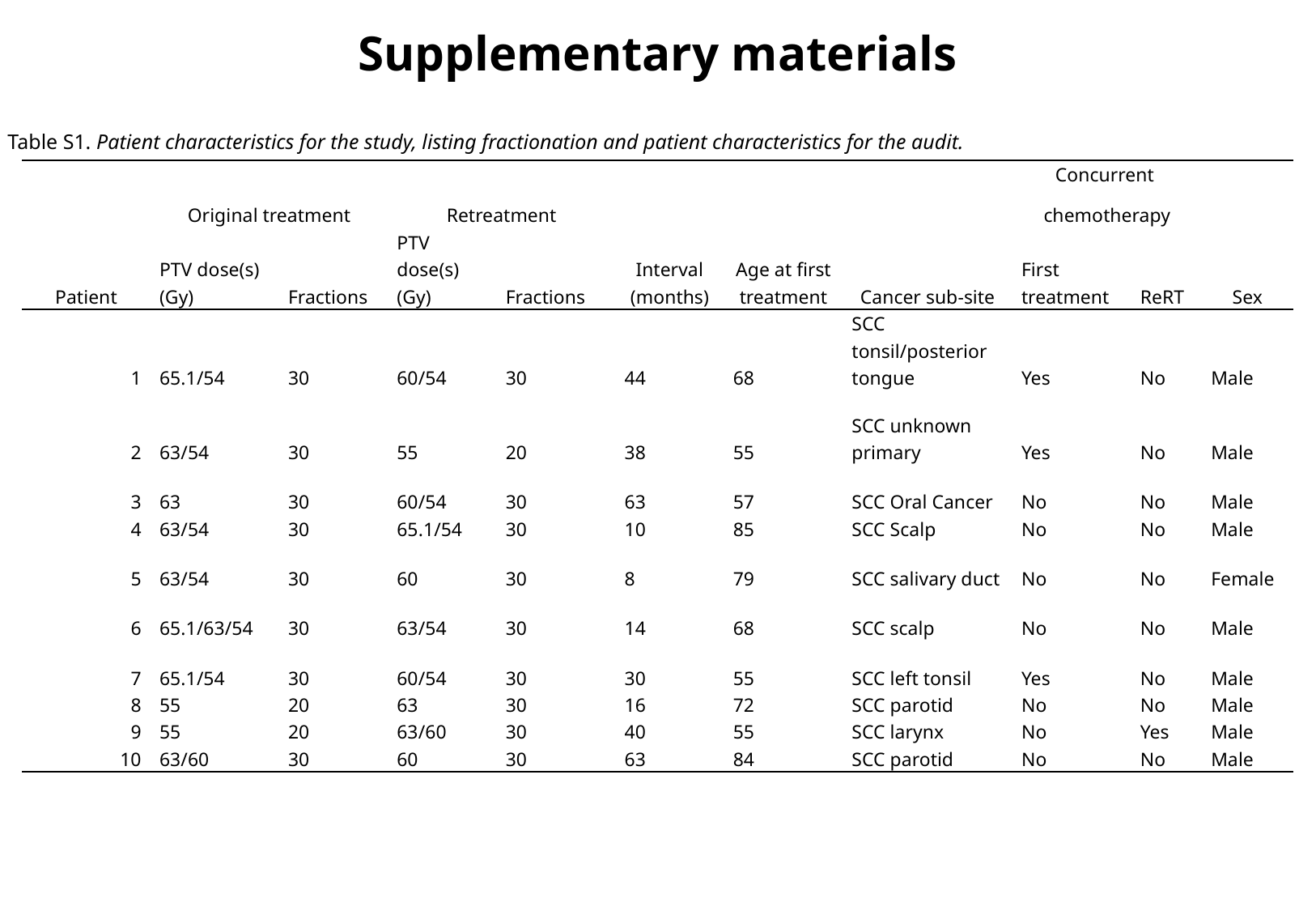

Supplementary materials
Table S1. Patient characteristics for the study, listing fractionation and patient characteristics for the audit.
| Patient | Original treatment | | Retreatment | | Interval (months) | Age at first treatment | Cancer sub-site | Concurrent chemotherapy | | Sex |
| --- | --- | --- | --- | --- | --- | --- | --- | --- | --- | --- |
| | PTV dose(s) (Gy) | Fractions | PTV dose(s) (Gy) | Fractions | | | | First treatment | ReRT | |
| 1 | 65.1/54 | 30 | 60/54 | 30 | 44 | 68 | SCC tonsil/posterior tongue | Yes | No | Male |
| 2 | 63/54 | 30 | 55 | 20 | 38 | 55 | SCC unknown primary | Yes | No | Male |
| 3 | 63 | 30 | 60/54 | 30 | 63 | 57 | SCC Oral Cancer | No | No | Male |
| 4 | 63/54 | 30 | 65.1/54 | 30 | 10 | 85 | SCC Scalp | No | No | Male |
| 5 | 63/54 | 30 | 60 | 30 | 8 | 79 | SCC salivary duct | No | No | Female |
| 6 | 65.1/63/54 | 30 | 63/54 | 30 | 14 | 68 | SCC scalp | No | No | Male |
| 7 | 65.1/54 | 30 | 60/54 | 30 | 30 | 55 | SCC left tonsil | Yes | No | Male |
| 8 | 55 | 20 | 63 | 30 | 16 | 72 | SCC parotid | No | No | Male |
| 9 | 55 | 20 | 63/60 | 30 | 40 | 55 | SCC larynx | No | Yes | Male |
| 10 | 63/60 | 30 | 60 | 30 | 63 | 84 | SCC parotid | No | No | Male |
